# Gpr18 agonist dampens inflammation, enhances myogenesis, and restores muscle function in models of Duchenne muscular dystrophy

**DOI:** 10.3389/fcell.2023.1187253

**Published:** 2023-08-14

**Authors:** Junio Dort, Zakaria Orfi, Melissa Fiscaletti, Philippe M. Campeau, Nicolas A. Dumont

**Affiliations:** ^1^ CHU Sainte-Justine Research Center, Montreal, QC, Canada; ^2^ School of Rehabilitation, Faculty of Medicine, Université de Montréal, Montreal, QC, Canada; ^3^ Department of Pharmacology and Physiology, Faculty of Medicine, Université de Montréal, Montreal, QC, Canada; ^4^ Department of Pediatrics, Faculty of Medicine, Université de Montréal, Montreal, QC, Canada

**Keywords:** muscle, inflammation, bioactive lipid, muscular dystrophies, muscle stem cell (MuSC)

## Abstract

**Introduction:** Muscle wasting in Duchenne Muscular Dystrophy is caused by myofiber fragility and poor regeneration that lead to chronic inflammation and muscle replacement by fibrofatty tissue. Our recent findings demonstrated that Resolvin-D2, a bioactive lipid derived from omega-3 fatty acids, has the capacity to dampen inflammation and stimulate muscle regeneration to alleviate disease progression. This therapeutic avenue has many advantages compared to glucocorticoids, the current gold-standard treatment for Duchenne Muscular Dystrophy. However, the use of bioactive lipids as therapeutic drugs also faces many technical challenges such as their instability and poor oral bioavailability.

**Methods:** Here, we explored the potential of PSB-KD107, a synthetic agonist of the resolvin-D2 receptor Gpr18, as a therapeutic alternative for Duchenne Muscular Dystrophy.

**Results and discussion:** We showed that PSB-KD107 can stimulate the myogenic capacity of patient iPSC-derived myoblasts *in vitro*. RNAseq analysis revealed an enrichment in biological processes related to fatty acid metabolism, lipid biosynthesis, small molecule biosynthesis, and steroid-related processes in PSB-KD107-treated mdx myoblasts, as well as signaling pathways such as Peroxisome proliferator-activated receptors, AMP-activated protein kinase, mammalian target of rapamycin, and sphingolipid signaling pathways. *In vivo*, the treatment of dystrophic mdx mice with PSB-KD107 resulted in reduced inflammation, enhanced myogenesis, and improved muscle function. The positive impact of PSB-KD107 on muscle function is similar to the one of Resolvin-D2. Overall, our findings provide a proof-of concept that synthetic analogs of bioactive lipid receptors hold therapeutic potential for the treatment of Duchenne Muscular Dystrophy.

## Introduction

Duchenne Muscular Dystrophy (DMD) is a severe disease caused by genetic variants in the *Dystrophin* gene. The disease is characterized by progressive muscle degeneration and wasting. Many factors contribute to disease progression such as myofiber fragility, chronic inflammation, accumulation of fibrofatty tissue, and muscle stem cell (MuSC) dysfunctions (Dumont et al., 2015; Filippelli and Chang, 2022). Glucocorticoids, the current gold-standard treatment for the disease, modulate the inflammatory response, which partially protects the muscle from degeneration and prolongs muscle function (Manzur et al., 2008). In acute conditions, glucocorticoids can promote the restorative phenotype of macrophages (Caratti et al., 2023) and muscle regeneration post-injury (Lin et al., 2021). However, chronic administration of glucocorticoids also has detrimental side effects on fibrofatty tissue accumulation, MuSC regenerative capacity, and muscle degradation/synthesis pathways (te Pas et al., 2000; Dong et al., 2013; Dort et al., 2021; Molina et al., 2021). These opposite effects of glucocorticoids could be mediated by the type of muscle injury, the timing of administration, the type of glucocorticoids, and/or the dosage (Quattrocelli et al., 2017). Nonetheless, there is room for the development of novel therapies that maximize the pro-resolving and pro-regenerative effects on muscle recovery.

Recent findings indicate that Resolvins, bioactive lipids biosynthesized from the conversion of the omega-3 fatty acids (e.g., docosahexaenoic acid or eicosapentaenoic acid) by the enzymes 5- and 15-lipoxygenase, have a potent capacity to promote muscle regeneration. Resolvin-D2 (RvD2) administration in cardiotoxin-injured muscle was shown to decrease inflammation and stimulate muscle function recovery (Giannakis et al., 2019). Similarly, Resolvin-D1 (RvD1) was shown to enhance muscle regeneration and function by modulating MuSC response and promoting the pro-regenerative phenotype of macrophages (Markworth et al., 2020). Moreover, administration of RvD1 is able to rejuvenate aged muscle regeneration by reducing the chronic inflammatory process, which restores muscle function (Markworth et al., 2021). Our recent findings indicate that RvD2 has a stronger therapeutic potential than glucocorticoids for the treatment of DMD (Dort et al., 2021). Resolvin-D2 dampens inflammation and directly targets myogenic cells to enhance their regenerative capacity and improve muscle function.

RvD2 has been shown to target the Gpr18 receptor, which has been proposed as a candidate cannabinoid receptor (Chiang et al., 2015). This receptor is expressed by myogenic cells during differentiation, and inhibition or deletion of this receptor ablates the effect of RvD2 on myogenic cells (Dort et al., 2021). These findings open a new therapeutic avenue aiming to target the Gpr18 receptor with synthetic agonists to stimulate muscle regeneration. One of the drawbacks to the use of bioactive lipids, such as RvD2, as a therapeutic approach is that they have a short-half life *in vivo* due to their rapid degradation or metabolization by enzymes (Giannakis et al., 2019; Krashia et al., 2019). Moreover, bioactive lipids are unstable and must be stored at −80°C, which complexifies their distribution and commercialization. Further, bioactive lipids are poorly absorbed in the gastrointestinal tract and have a low bioavailability, which could be optimized by the use of nanoparticle carriers to enhance the efficacy of oral delivery (Lin et al., 2017). To the best of our knowledge, the efficacy of the oral delivery of RvD2 has not been investigated; however, a few studies have shown resolvin-D1 have therapeutic effects when administered orally (Recchiuti et al., 2014; Codagnone et al., 2018; Mottola et al., 2020).

Another approach to improve the clinical transferability of bioactive lipids would be the use of stable synthetic analogs. Contrarily to bioactive lipids, synthetic analogs are easier to store and could be provided as crystalline form, which is more convenient for commercialization. In the recent years, synthetic analogs with high affinity and specificity for Gpr18 have been developed (Schoeder et al., 2020). These molecules, such as PSB-KD107, have high agonistic activity for Gpr18 and low affinity to other cannabinoid receptors such as CB1, CB2, and Gpr55. Here, we investigated the therapeutic potential of the Gpr18 agonist, PSB-KD107, for the treatment of DMD. Using induced-pluripotent stem cells (iPSC)-derived myoblasts generated from samples of patients with muscular dystrophies, we showed that Gpr18 agonist can stimulate myogenesis of human cells *in vitro*. Transcriptomics analysis revealed that biological processes related to fatty acid biosynthesis, and pathways such as Peroxisome proliferator-activated receptors (PPAR) and AMP-activated protein kinase (AMPK) are enriched in PSB-KD107-treated cells. Treatment of dystrophic mdx mice with PSB-KD107 resulted in a reduction of muscle inflammation and an increase in the myogenic cell pool compared to control. These changes resulted in an improvement in muscle function that is similar to the one induced by RvD2. Overall, these findings support the therapeutic potential of Gpr18 agonist for the treatment of DMD.

## Results

**FIGURE 1:**
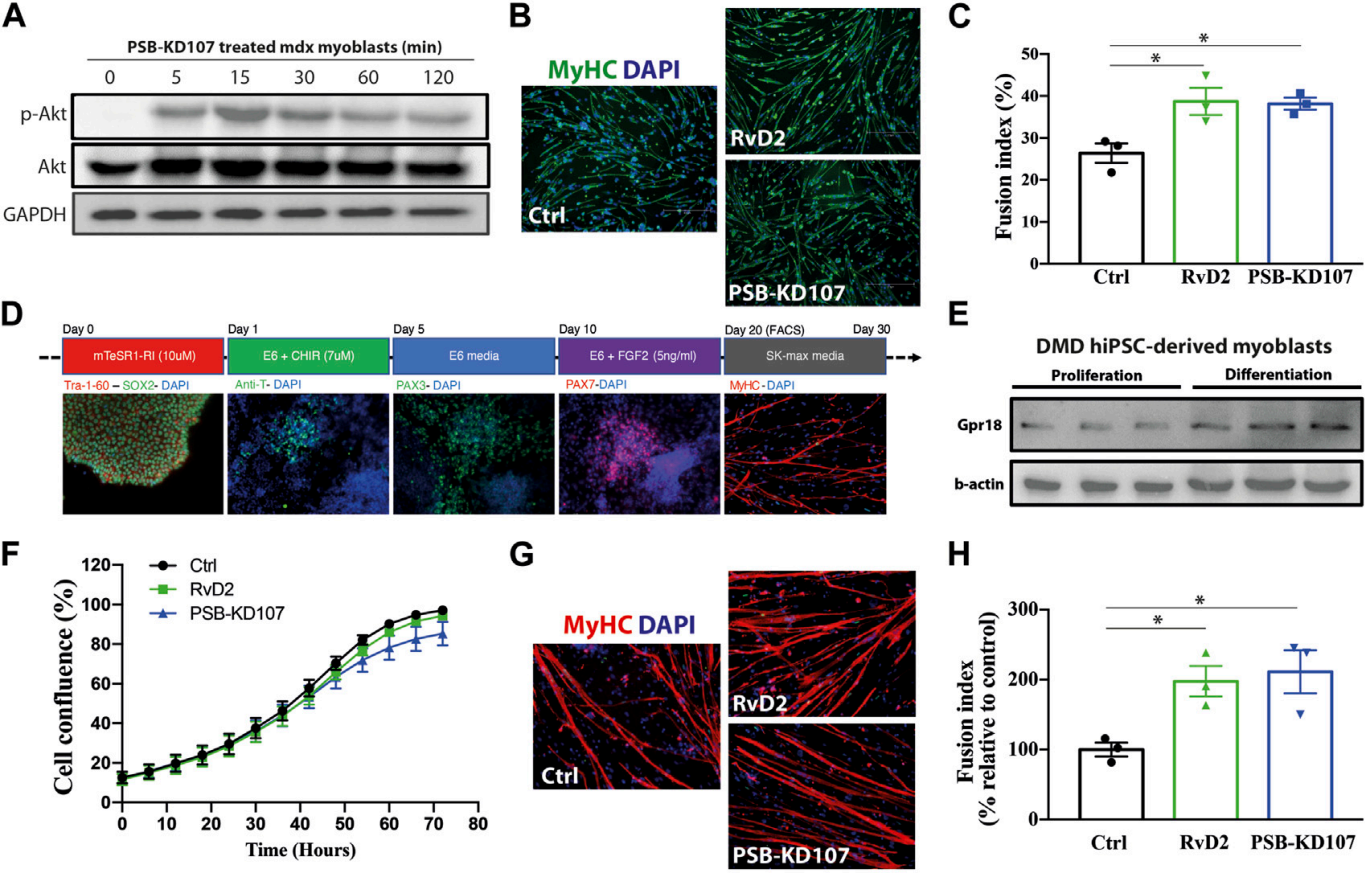
Effect of Gpr18 agonist on mouse and human myogenesis. **(A)** Western blot on mdx myoblasts treated with PSB-KD107 (10 μM) for 0, 5, 15, 30, 60, 120 min showing the expression of p-Akt, total Akt, and GAPDH (experiment performed in biological duplicates with similar results). **(B)** Representative micrographs of Myosin Heavy chain (MyHC, green) and DAPI (nuclei, blue), and **(C)** quantification of fusion index in mdx myoblasts differentiated 4 days and treated with Resolvin-D2 (RvD2; 200 nM), PSB-KD107 (10 μM), or vehicle. n = 3 independent biological samples from different mdx mice. **(D)** Representative micrographs showing the time course differentiation of hiPSC into myogenic cells. **(E)** Western blot showing the expression of GPR18 in proliferating and differentiating DMD hiPSC-derived myoblasts (experiments performed in technical duplicates with similar results). **(F)** Proliferation curve of DMD hiPSC-derived myoblasts treated with PSB-KD107, RvD2, or vehicle. **(G)** Representative micrographs of MyHC (red) and DAPI (blue), and **(H)** quantification of the fusion index of DMD hiPSC-derived myoblasts differentiated 7 days and treated with PSB-KD107, RvD2, or vehicle. n = 3 independent biological samples from different patients. Results shown as mean+/- SEM. *p < 0.05 (One-Way ANOVA with Fisher LSD *post hoc* test).

We previously demonstrated that Gpr18 is expressed in mouse myoblasts and that RvD2 activates the Akt signaling pathway in these cells, and stimulates myoblast differentiation/fusion (Dort et al., 2021). To determine if the Gpr18 agonist could mimic these effects, primary myoblasts from mdx mice were treated with PSB-KD107 and the activation of the Akt pathway was assessed at different time points (0, 5, 15, 30, 60, 120 min). PSB-KD107 administration induced a rapid Akt phosphorylation that peaked 15–30 min after the treatment (Figure 1A; Supplementary Figure S1A). Moreover, treatment of differentiating myoblasts for 4 days with PSB-KD107 increased the fusion index at a similar level to the one induced by RvD2 (Figures 1B, C). Thereafter, to determine the translational potential of the Gpr18 agonist, we assessed its capacity to target human myogenic cells. To do so, we used a model of human induced pluripotent stem cells (hiPSC)-derived myoblasts generated from blood samples collected from patients with DMD or Becker Muscular Dystrophy (BMD) (Figure 1D). The expression of Gpr18 was detected in proliferating hiPSC-derived myoblasts and was increased during differentiation (Figure 1E; Supplementary Figure S1B). Like RvD2, the addition of PSB-KD107 to the medium of proliferating hiPSC-derived myoblasts did not affect cell expansion (Figure 1F). On the other hand, the treatment of differentiating myoblasts with PSB-KD107 increased myoblast fusion into multinucleated myotubes compared to vehicle-treated cells (Figures 1G, H). This effect was similar to the one induced by RvD2.

**FIGURE 2:**
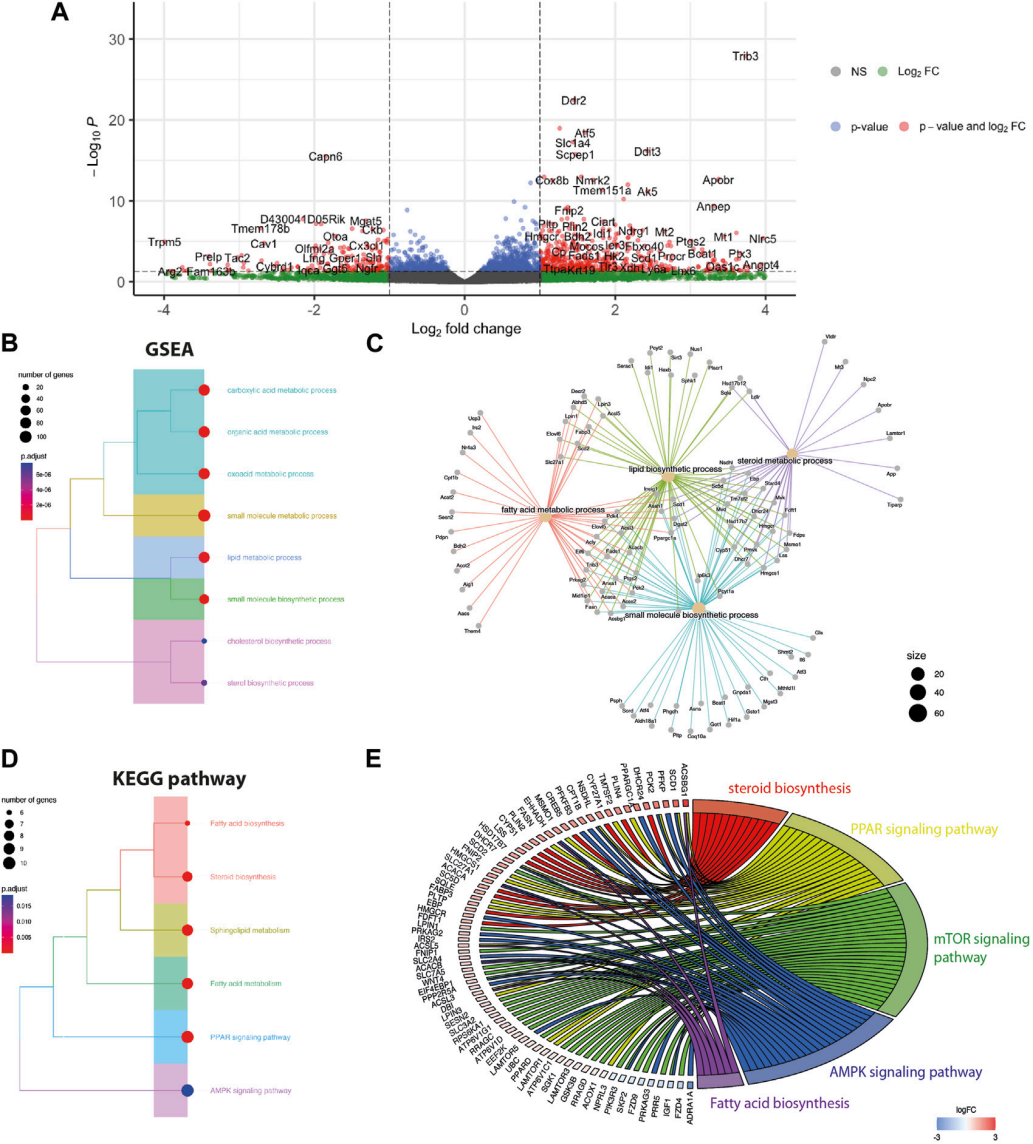
Effect of Gpr18 agonist on transcriptomic changes of myoblasts. **(A–E)** RNA seq analysis on mdx myoblasts treated with PSB-KD107 (10 μM) or vehicle. **(A)** Volcano plots showing the top upregulated and downregulated genes in PSB-KD107-treated cells compared to vehicle. **(B,D)** Tree plot showing gene set enrichment analysis (GSEA) **(B)**, or KEGG pathway enrichment **(D)** of the differentially expressed genes (upregulated by PSB-KD107) that were grouped according to functional terms. **(C,E)** Cnet plot of GSEA and KEGG showing the interacting network between genes, biological processes, and pathways.

To determine the effect of the Gpr18 agonist on cell transcriptome and signaling, a RNAseq analysis was performed on mdx myoblasts treated with PSB-KD107 or vehicle overnight (16 h). A total of 385 genes were differentially expressed (p-adj <0.05), including 296 genes upregulated and 89 genes downregulated (Figure 2A). GO term analysis revealed that the top downregulated genes in PSB-KD107-treated cells were related to RNA splicing (Supplementary Figure S2). On the other hand, enrichment analysis of the top upregulated genes (>2-fold change) identified that biological processes such as lipid and fatty acid metabolism, small molecule biosynthesis, and steroid-related processes were enriched in PSB-KD107-treated cells (Figure 2B). Cnet plot showed that there is a strong interaction network between the genes and the enriched biological concepts (Figure 2C). KEGG enrichment analysis identified that pathways such as Peroxisome proliferator-activated receptors (PPAR), AMP-activated protein kinase (AMPK), mammalian target of rapamycin (mTOR) and sphingolipid signaling pathways are activated by the treatment with the Gpr18 agonist (Figure 2D). Cnet plot revealed that there is a strong interacting network between these different signaling pathways (Figure 2E).

**FIGURE 3:**
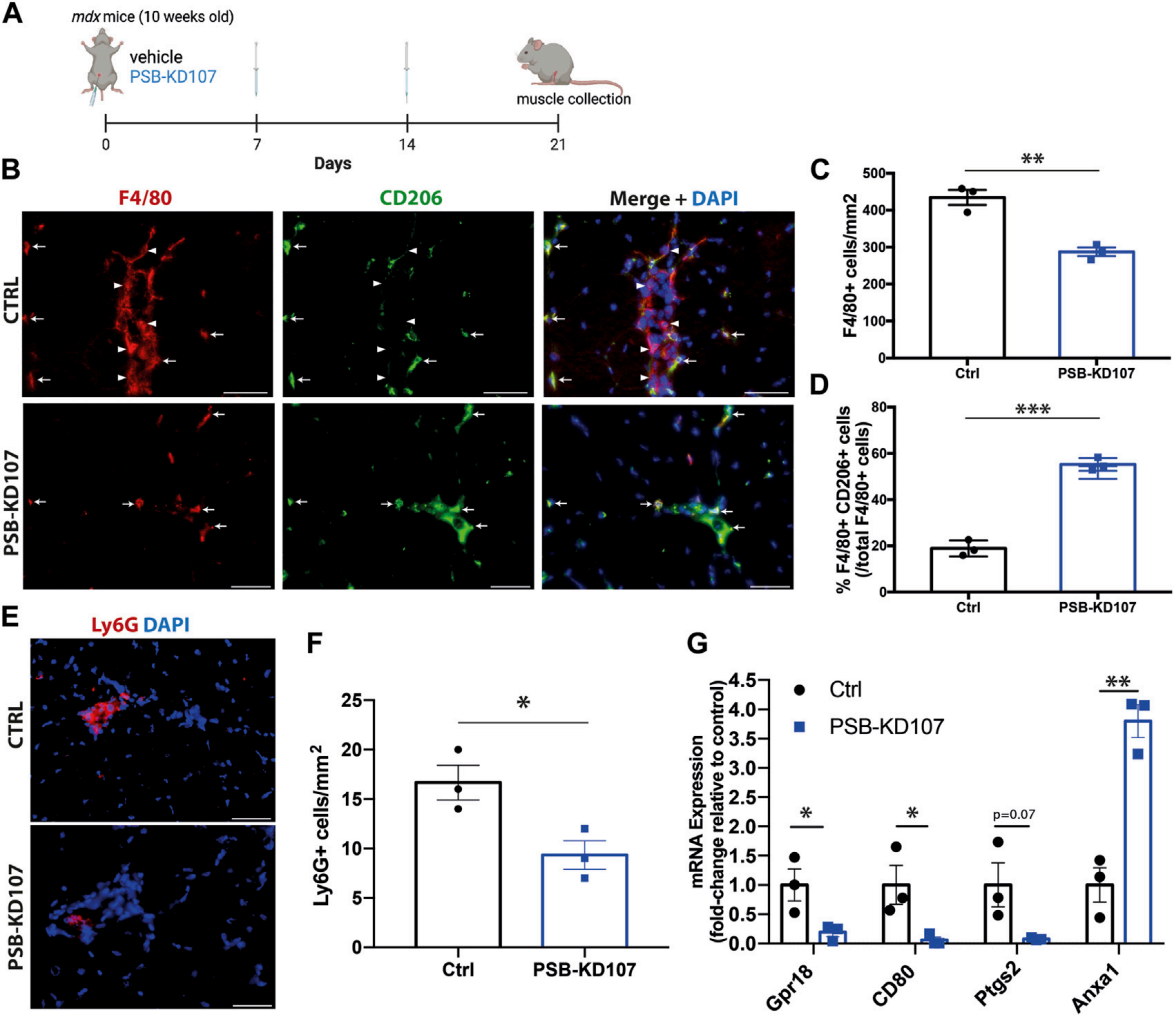
Effect of Gpr18 agonist on inflammation in dystrophic mice. **(A)** Schematic showing the treatment of mdx mice with weekly injections of PSB-KD107 (1 mg/kg) or vehicle for 3 weeks (image created with biorender.com). **(B)** Representative immunofluorescence for the pan-macrophage marker (F4/80, red), the anti-inflammatory macrophage marker (CD206, green), and DAPI (nuclei, blue). White arrowheads indicate pro-inflammatory macrophages (F4/80^+^CD206^-^) and white arrows show anti-inflammatory macrophages (F4/80^+^CD206^+^). Scale bars = 50 μm. **(C)** Quantification of the total number of macrophages, and **(D)** the proportion of anti-inflammatory macrophages (F4/80^+^CD206^+^ cells/total F4/80^+^ cells) in PSB-KD107 and vehicle-treated (Ctrl) mice. **(E)** Representative immunofluorescence for the neutrophil marker Ly6G (red) and DAPI (nuclei, blue). **(F)** Quantification of the neutrophil count in PSB-KD107 and vehicle-treated (Ctrl) mice. Scale bars = 50 μm. **(G)** qPCR showing the expression of pro-inflammatory genes (*CD80, Gpr18, Ptgs2*) and anti-inflammatory gene (*Anxa1*) in skeletal muscle of mdx mice treated with PSB-KD107 or vehicle. N = 3. Results shown as mean+/- SEM. *p < 0.05, **p < 0.01, ***p < 0.001 (Unpaired *t*-test).

To assess the therapeutic potential of Gpr18 agonist *in vivo*, mdx mice were treated weekly with PSB-KD107 or vehicle (Figure 3A). Immunofluorescence was performed on sections of the tibialis anterior muscle of the treated mice to assess inflammatory markers. The results showed that PSB-KD107 decreased total macrophage density by ∼35% (Figures 3B, C). Moreover, analysis of the different subsets of macrophages revealed that PSB-KD107 increases by 2.9-fold the proportion of macrophages expressing the anti-inflammatory marker CD206 (Figures 3B, D). Immunofluorescence for the neutrophil marker Ly6G, also revealed a ∼45% reduction in the neutrophil count in PSB-KD107 treated mice (Figures 3E, F). qPCR analysis of skeletal muscle homogenates was performed to assess the expression of pro- and anti-inflammatory markers that were previously identified to be affected by RvD2 treatment (Dort et al., 2021). qPCR analysis revealed that the expression of the pro-inflammatory markers *Cd80, Gpr18, and Ptgs2* was decreased by PSB-KD107 treatment, while the expression of the anti-inflammatory marker *Anxa1* was increased by the treatment with PSB-KD107 compared to control (Figure 3G).

**FIGURE 4:**
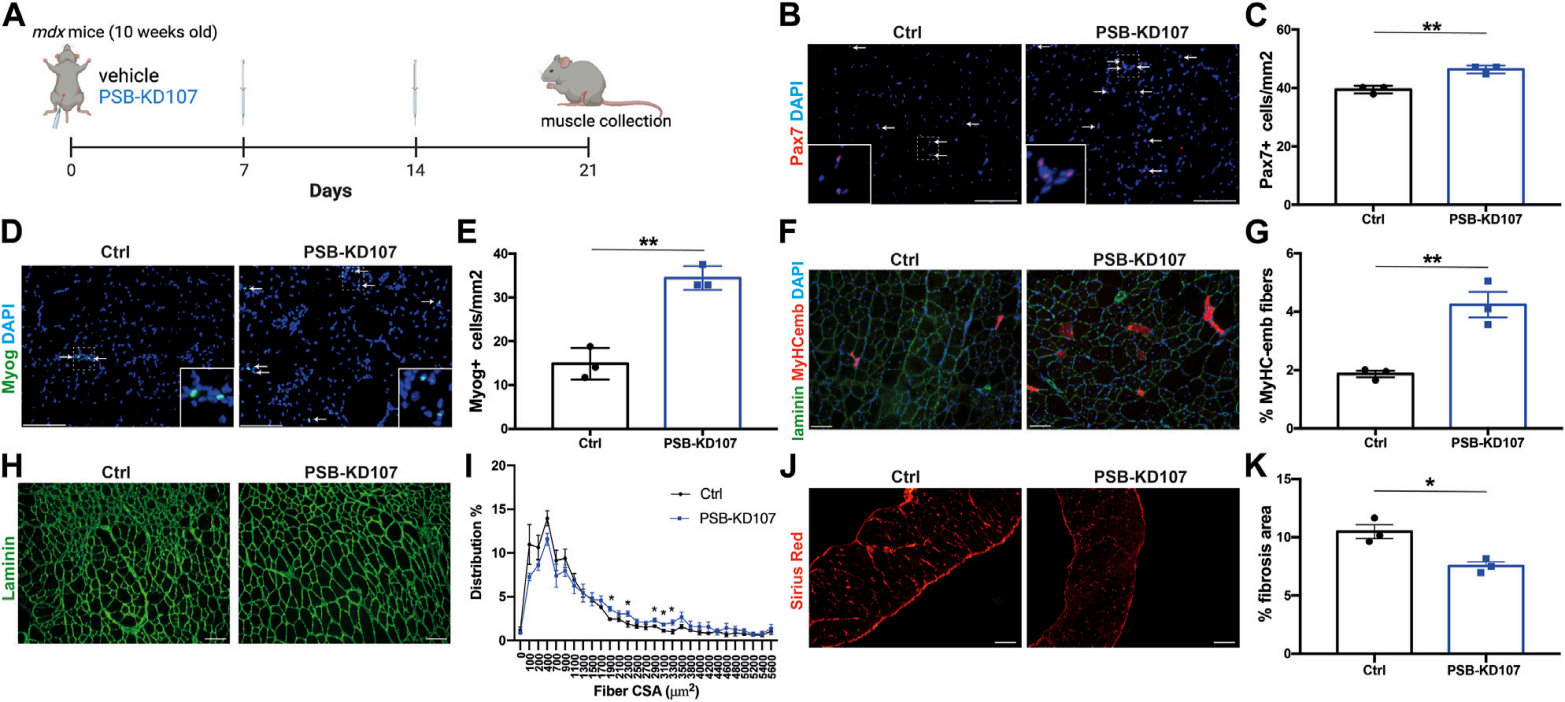
Effect of Gpr18 agonist on myogenesis in dystrophic mice. **(A)** Schematic showing the treatment of mdx mice with weekly injections of PSB-KD107 (1 mg/kg) or vehicle for 3 weeks (image created with biorender.com). **(B)** Representative micrograph showing Pax7 (MuSC marker; red) and DAPI (nuclei, blue) staining. **(C)** Quantification of Pax7^+^ cells in the muscle of PSB-KD107 or vehicle-treated mice. **(D)** Representative micrograph showing Myogenin (Myog, differentiated myoblast marker; green) and DAPI staining. **(E)** Quantification of Myog^+^ cells in the muscle of PSB-KD107 or vehicle-treated mice. **(F)** Representative micrograph showing embryonic myosin heavy chain (MyHCemb, newly formed myofiber marker; red) and laminin (to delineate myofiber border; green), and DAPI staining. **(G)** Quantification of the proportion of MyHCemb^+^ myofibers (% of total myofibers) in PSB-KD107 or vehicle-treated mice. **(H)** Representative micrograph showing laminin (green) staining, and **(I)** quantification of fiber cross-sectional area (CSA) in the TA muscle of PSB-KD107 or vehicle-treated mice. **(J)** Representative micrograph showing Sirius red staining visualized under polarized light (red, collagen marker), and **(K)** quantification of fibrosis area in the diaphragm muscle of PSB-KD107 or vehicle-treated mice. Scale bars = 100 μm. N = 3-4. Results shown as mean+/- SEM. *p < 0.05, **p < 0.01 (Unpaired *t*-test).

Next, we assessed the impact of PSB-KD107 on the myogenic cell pool and on muscle regeneration in treated mdx mice (Figure 4A). Immunofluorescence analysis of Pax7^+^ cells revealed a modest (15%–20%) but significant increase in the number of MuSC in PSB-KD107 treated mice (Figures 4B, C). The analysis of Myog^+^ cells showed a stronger increase (2.4-fold) in the number of differentiated myoblasts (Figures 4D, E). These changes were associated with a 2.3-fold increase in the number of newly-formed myofibers expressing embryonic myosin heavy chain (MyHC-emb) (Figures 4F, G). The assessment of myofiber cross-sectional area revealed a shift in fiber size frequency distribution toward higher number of larger myofibers in the TA muscle of mice treated with PSB-KD107 compared to vehicle (Figures 4H, I), although this change did not result in a significant increase of the mean myofiber cross-sectional area (Supplementary Figure S3). Finally, to determine if these improvements in the regenerative profile were associated with changes in fibrosis level, we performed Sirius-red staining on sections of the diaphragm muscle (most severely affected muscle in mdx mouse). Our findings show that PSB-KD107 reduces muscle fibrosis by ∼25% compared to vehicle (Figures 4J, K).

**FIGURE 5:**
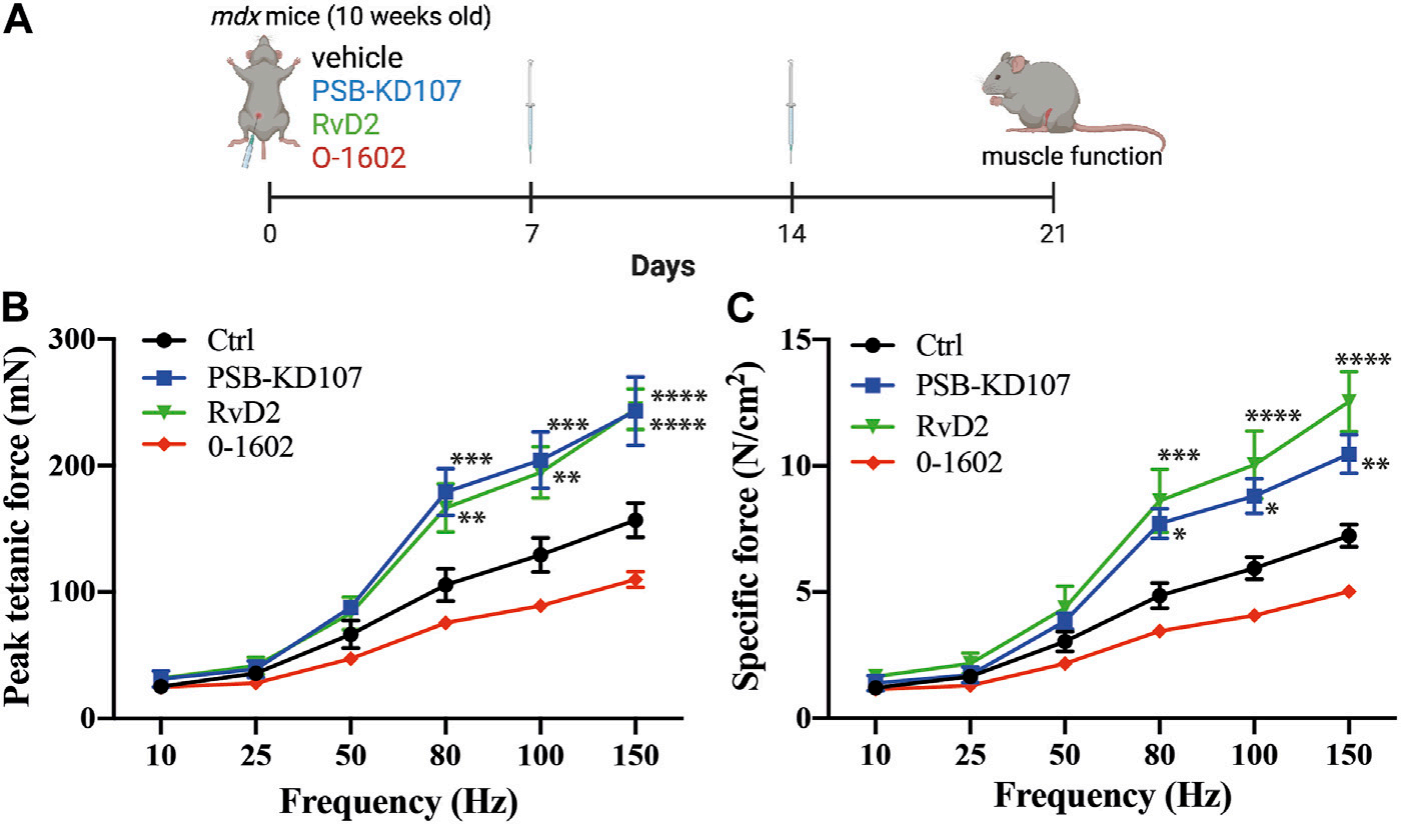
Effect of Gpr18 agonist on muscle function of dystrophic mice. **(A)** Schematic showing the treatment of mdx mice with weekly injections of PSB-KD107 (1 mg/kg), Resolvin-D2 (RvD2, 5 ug/kg), O-1602 (Gpr55 agonist, 5 mg/kg) or vehicle for 3 weeks (image created with biorender.com). **(B,C)** Effect of PSB-KD107 injection (blue line), Resolvin-D2 (green line), O-1602 (red line), or vehicle (black line) on absolute muscle force (mN) or specific muscle force (N/cm2) of mdx mice. N = 5 (except n = 3 for O-1602). Results shown as mean+/- SEM. *p < 0.05, **p < 0.01, ***p < 0.001, ****p < 0.0001 significantly different from control (2-way ANOVA followed by Tukey *post hoc* test).

To determine the therapeutic potential of Gpr18 agonist for the treatment of DMD, we assessed muscle function, which is a more clinically relevant outcome. Mdx mice were injected with PSB-KD107, RvD2, or vehicle (Figure 5A). Treatment with PSB-KD107 increased the absolute (mN) and the specific (N/cm^2^) maximal muscle force compared to controls (Figures 5B, C). The effect of PSB-KD107 was similar to the one of RvD2 (Figures 5B, C).

Gpr18, as well as other orphan receptors such as Gpr55, are considered as candidate cannabinoid receptors. Our previous findings demonstrated that the effect of RvD2 on dystrophic mice is ablated by O-1918, a selective non-CB1/CB2 receptor antagonist (Dort et al., 2021). This antagonist was shown to target Gpr18, but it could also inhibit the activity of Gpr55 (Morales et al., 2020). While PSB-KD107 is selective for Gpr18 over other cannabinoid receptors such as Gpr55, we aimed to determine if targeting the Gpr55 receptor could mimic the beneficial effect of Resolvin-D2. Therefore, we assessed the impact of O-1602, a Gpr55 agonist, on dystrophic mdx mice. Contrarily to PSB-KD107, treatment with O-1602 did not significantly affect muscle function in mdx mice (Figures 5B, C).

## Discussion

In this paper, we investigated the therapeutic potential of a strategy targeting the Gpr18 receptor for the treatment of DMD. Our findings show that the Gpr18 receptor is expressed in human myogenic cells and that it can be targeted by the agonist PSB-KD107 to enhance myogenesis. *In vivo* results demonstrate that this Gpr18 agonist reduces inflammatory markers and promotes the pro-resolving macrophage subset. This change in macrophage proportion could be mediated by the reduced infiltration of circulating pro-inflammatory monocytes, and/or by the stimulation of their phenotype switch, like what has been shown by RvD2 treatment (Spite et al., 2009; Dort et al., 2021). Treatment with PSB-KD107 enhances signs of myogenesis and induces a shift in myofiber size distribution, which is accompanied by an improvement in muscle function of dystrophic mice (similar to the one induced by RvD2). These results do not exclude that PSB-KD107 could mediate its therapeutic effects on muscle function through multiple mechanisms (e.g., excitation-contraction coupling, metabolism, fiber typing). These new findings provide a proof-of-concept that, like bioactive lipids, synthetic agonists could be used to improve the therapeutic applicability for the treatment of muscular dystrophies.

Gpr18 is mostly known for its involvement in the regulation of the immune response. It was shown in *Gpr18*-knockout mice that this receptor regulates the CD8 T cell compartment (Wang et al., 2014; Sumida and Cyster, 2018). However, Gpr18 was shown to be expressed in a variety of cells and tissues in humans and/or mice such as spleen, testis, heart, gastrointestinal tract, blood vessels, immune cells, and skeletal muscle (Gantz et al., 1997; Kohno et al., 2006; Takenouchi et al., 2012; MacIntyre et al., 2014; Matouk et al., 2017; Simcocks et al., 2020; Dort et al., 2021; Zhao et al., 2023). Therefore, the activation of the Gpr18 receptor could trigger a multitude of biological processes. For instance, *Gpr18*-deficient mice also showed defects in reperfusion following hindlimb muscle ischemia, indicating that this receptor also plays a role in revascularization (Zhang et al., 2016). Our findings indicate that the activation of Gpr18 stimulates muscle regeneration, although this effect can be mediated by a complex cellular response.

Many endogenous ligands of Gpr18 have been reported, namely, RvD2, N-arachidonoyl glycine (NAGly), and Δ9-tetrahydrocannabinol (THC). Specific binding of RvD2 on Gpr18 was shown *in vitro* (Chiang et al., 2015), although these results remain to be validated by an independent laboratory (Schebb et al., 2022). Nonetheless, the knockdown or knockout of this receptor was shown to ablate the effect of RvD2 *in vitro* and *in vivo* (Chiang et al., 2015; 2017; Zhang et al., 2016; Dort et al., 2021), and many independent studies have shown that the Gpr18 antagonist, O-1918, blocks the effect of RvD2 in various inflammatory conditions (Zuo et al., 2018; Lopategi et al., 2019; Dort et al., 2021; Lu et al., 2021; Bardin et al., 2022). The development of novel agonists, such as PSB-KD107, that have a stronger activation capacity compared to the endogenous ligands of Gpr18, opened new therapeutic opportunities (Schoeder et al., 2020). PSB-KD107 does not bind or activate other receptors such as Gpr55 or cannabinoid receptors at high concentration (10 uM) (Schoeder et al., 2020). Our findings indicate that PSB-KD107 activates p-Akt signaling similar to other Gpr18 ligands (Offertáler et al., 2003; Matouk et al., 2017; Dort et al., 2021). The activation of the Akt pathway was also observed by agonists of other resolvin receptors such as the Resolvin-E1 receptor ChemR23 (Trilleaud et al., 2021). This activation of the Akt pathway is consistent with our RNAseq dataset showing that PSB-KD107 induces the expression of genes related to the mTOR pathway.

Gpr18 agonists have been used for the treatment of different conditions. For instance, the administration of PSB-KD107 was shown to induce vasodilation, and affect blood pressure and heart function in the short term (Kotańska et al., 2021). Similarly, other synthetic analogs of Gpr18 such as PSB-MZ-1415 and PSB-MZ-1440 showed the same vasorelaxation activity (Kozłowska et al., 2022). Another Gpr18 synthetic analog, PSB-KK-145, was shown to reduce inflammatory markers and signs of pain in models of colitis (Fabisiak et al., 2021). Our findings add to this growing literature by showing that Gpr18 can also reduce the inflammatory process in a model of chronic muscle degeneration and increase muscle regeneration and function. However, the activation of the Gpr18 receptor can also stimulate pathophysiological processes under specific conditions. For instance, Gpr18 and RvD2 were shown to be upregulated in human coronary arteries in presence of atherosclerotic lesions; and the treatment of ApoE-deficient hyperlipidemic mice with the Gpr18 antagonist O-1918 was shown to reduce atherosclerosis (Bardin et al., 2022). These results indicate that while Gpr18 agonists could have beneficial effects on degenerative and/or inflammatory conditions such as DMD, they could also contribute to the pathogenesis of other conditions, which should be carefully monitored.

Our RNAseq dataset revealed that PSB-KD107 administration to dystrophic myoblasts *in vitro* activates biological processes related to the fatty acid metabolism. Changes in metabolism have been described to control myogenic transition by triggering histone acetylation and the regulation of muscle gene transcription (Ryall et al., 2015; Yucel et al., 2019). The transcriptomic changes induced by PSB-KD107 in the biosynthesis of fatty acids, sphingolipids, cholesterol, and steroids, are highly coherent with the activation of a bioactive lipid receptor, and they correlate well with the phenotype observed *in vivo* of reduced inflammation and enhanced regeneration. For instance, sphingolipids such as sphingosine-1-phosphate stimulate myoblast differentiation and myotube growth; and ameliorate muscle phenotype in a model of DMD (Pantoja et al., 2013; Tan-Chen et al., 2020). Similar changes in the fatty acid metabolism were also observed in a previous study on obese rats, which showed that administration of the Gpr18 agonist PSB-KK-1415 reduced weight gain and increased the expression of high-density lipoprotein (without affecting the total cholesterol level) (Kotańska et al., 2021). High-density lipoproteins were shown to decrease the production of the pro-inflammatory lipid leukotriene-B4, and increase the production of the pro-resolving lipids lipoxin-B4 and resolvin-E2 by macrophages (Tsuda et al., 2017). Therefore, Gpr18 agonists could potentially induce a positive feed-back loop leading to the biosynthesis of pro-resolving mediators.

KEGG analysis of our RNAseq dataset revealed that genes related to the mTOR, AMPK and PPAR signaling pathways are enriched in PSB-KD107-treated cells. Our findings and others have shown that Resolvins activate the Akt/mTOR pathway (Maekawa et al., 2015; Dort et al., 2021). This pathway has been well described to play an important role in myoblast differentiation and fusion (Wilson and Rotwein, 2007; Gardner et al., 2012), which is coherent with the pro-differentiating effect that we observed following PSB-KD107 treatment *in vitro* and *in vivo*. Resolvins were also shown to induce the activation of AMPK in macrophages and increase fatty acid oxidation and efficient phagocytosis (Hosseini et al., 2021; Chiang et al., 2022). The activation of the AMPK is essential to induce macrophage switch toward their anti-inflammatory phenotype during muscle regeneration (Mounier et al., 2013; Caratti et al., 2023). Activation of AMPK by the binding of Annexin A1 to the Fpr2 (formylpeptide receptor 2) receptor (which is also targeted by the bioactive lipid lipoxin-A4) was shown to regulate macrophage skewing, inflammation resolution, and muscle regeneration (McArthur et al., 2020). Moreover, it was shown that AMPKα1 controls MuSC self-renewal by regulating its metabolism through the activity of lactate dehydrogenase (Theret et al., 2017). Similarly, PPARγ plays a central role in the polarization of anti-inflammatory macrophages, which stimulate myoblast fusion and muscle regeneration by the release of factors such as Growth Differentiation Factor-3 (GDF3) (Odegaard et al., 2007; Varga et al., 2016). These results are consistent with the increase in the pro-resolving macrophage subset that we observed in PSB-KD107-treated mdx mice.

By comparing the differentially expressed genes upregulated in PSB-KD107-treated myoblasts to another RNAseq dataset in which bone marrow derived macrophages were treated with Resolvin-D2 (Giannakis et al., 2019), we observed that many genes upregulated by PSB-KD107 treatment were also found in the top 75 upregulated genes induced by Resolvin-D2, such as *Gpr35, Pde3b, Trib3, Chac1, Nr4a1, Lipg, Ankrd37*, and *Kbtbd11*. Notably, *Trib3* (the top upregulated gene in our dataset) can inhibit the pro-inflammatory signaling pathway NF-kB by interacting with RelA (Wu et al., 2003; Duggan et al., 2010). Similarly, *Nr4a1* (*Nur77*) can also inhibit NF-kB signaling and play a key role in macrophage transition to the anti-inflammatory phenotype (Li et al., 2017; Koenis et al., 2018). This transcription factor has also been shown to promote myogenesis and muscle growth (Cortez-Toledo et al., 2017). Overall, our transcriptomics analysis is coherent with the literature and with our *in vivo* findings showing that PSB-KD107 stimulates the anti-inflammatory macrophage phenotype and muscle regeneration in mdx mice.

Our findings indicate that the Gpr55 agonist cannot mimic the beneficial effect of Gpr18 agonist in mdx mice. The Gpr55 agonist O-1602 was shown to modulate pain and inflammation in immune disorders (Zhou et al., 2016). However, treatment of C2C12 myoblasts with O-1602 had no effect on key pathways important for skeletal muscle energy homeostasis, oxidative capacity, and fatty acid metabolism (Simcocks et al., 2020). These results suggest that contrary to Gpr18 agonists, this Gpr55 agonist cannot efficiently target myogenic cells to stimulate myogenesis.

Overall, our findings provide a proof-of-concept that Gpr18 agonists can be used as an alternative therapeutic strategy to bioactive lipids, which have low stability and bioavailability. Our results indicate that Gpr18 agonist activates fatty acids metabolism/biosynthesis and signaling pathways that plays a key role in the resolution of inflammation. Our findings also indicate that Gpr18 agonists can target myogenic cells to stimulate myogenesis. Further studies are needed to explore the clinical applicability of this approach.

## Methods

### Animal husbandry

Male *mdx* mice (C57BL/10, Jackson Laboratory; Bar Harbor, ME) at ten weeks-of-age were used to reduce the variability associated with the initial disease onset phase (Spurney et al., 2009). Mice were individually housed on a 12:12 h light:dark cycle at the animal facility of the CHU Sainte-Justine research center. They were randomly assigned to four experimental groups, which received a weekly *i.p.* injection of either vehicle (DMSO diluted in aqueous buffer), Gpr18 agonist (PSB-KD107, 1 mg/kg, Cayman Chemical), resolvin D2 (RvD2, 5 μg/kg, Cayman Chemical) (Dort et al., 2021), or Gpr55 agonist (O-1602, 5 mg/kg, Cayman Chemical) (Walsh et al., 2015; Simcocks et al., 2020) over 3 weeks. Mice had free access to food and tap water. At the end of the experimental period, the extensor digitorum longus, tibialis anterior, and diaphragm muscles were collected to measure the contractile properties or muscle composition. Muscles were flash frozen in liquid nitrogen or embedded in OCT freezing medium (Fisher Scientific) and frozen in 2-methylbutane cooled in liquid nitrogen. Mice were anesthetized with pentobarbital sodium (50 mg/kg), followed by cervical dislocation. This study was approved by the CHU Sainte-Justine Research Ethics Committee and followed the guidelines of the *Comité Institutionnel des Bonnes Pratiques Animales en Recherche* (CIBPAR; approval number 2022-3608) in accordance with the Canadian Council on Animal Care guidelines.

### Participants’ recruitment, sample collection, and hiPSC-derived myoblasts generation

Patients and/or parents gave their written consent for blood sample collection, in accordance with the protocol approved by the CHU Sainte-Justine institutional review board (approval number 2015-853, 4072). Two of the patients were diagnosed with DMD and one was diagnosed with BMD. The peripheral blood mononuclear cells were reprogrammed into hiPSCs at the CHU Sainte-Justine stem cell core facility using integration-free based Sendai virus (Moquin-Beaudry et al., 2022). Following transduction, emerging clones were manually picked and cultured under feeder-free conditions using Matrigel-coated dishes and mTeSRPlus for a minimum of 15 passages to ensure stable pluripotency. Cells were characterized and showed a normal karyotype and expressed the human SSEA-4, Sox2, OCT4 and TRA1-60 makers. The differentiation of hiPSCs into myogenic progenitor cells was performed using a previously described transgene-free protocol (Shelton et al., 2016; Fabre et al., 2022). Briefly, 1 day prior differentiation, hiPSCs were gently dissociated with TrypLE™(Gibco, #12604013), and 10^5^ cells/well were plated as small colonies (10-30 cells/colony) on a Vitronectin-coated 12-well plates. The cells were cultured in mTeSR1 media (STEMCELL Technologies, #85850) supplemented with 10 μM ROCK inhibitor overnight (Y-27632, STEMCELL Technologies, #72302). Next day, the TeSR-E6 media (STEMCELL Technologies, #05946) supplemented with 7 μM CHIR99021 (STEMCELL Technologies, #72052) was added to the small colonies for 3 days. Thereafter, cells were gently washed with DPBS and cultured in TeSR-E6 medium (changed every day). At this stage, the cells form embryonic bodies, and start to express somite markers such as PAX3 and MEOX1. At day 10 of differentiation, the myogenic progenitor cells emerge from the somite-like structures, and the TeSR-E6 medium is supplemented with 5 ng/mL of FGF2 (Wisent Inc., #511-126-QU) to support their proliferation. At day 20, the cells start to express the MuSC marker PAX7. At this stage, the myogenic cells are purified by FACS (see next section). The differentiation of hiPSC-derived myoblasts into myotubes was performed by incubating the myoblasts at ∼80% confluence with differentiation medium consisting of DMEM (Wisent, 319-010-CL) supplemented with 1% Penicillin-Streptomycin (Thermo Fisher scientific, # 15070063), and 5% Horse Serum (Gibco, #16050130) for 7 days. During the experimental protocol, the medium was supplemented with RvD2 (200 nM), PSB-KD107 (10 μM) or vehicle (DMSO) from the first day of differentiation to the day 7, and the medium was renewed every 48 h.

### FACS and cell culture

For hiPSC-derived myoblasts, the cells are purified by FACS using the positive marker CD56-FITC (#562794, BD Biosciences), and the negative marker CD57-APC (#130-118-665, Miltenyi Biotech). For MuSC isolation from skeletal muscles of dystrophic *mdx* mice, the cells were purified by FACS using a negative selection for the FITC-conjugated antibodies anti-Sca-1 (Clone D7; 1:30; Miltenyi Biotech), anti-CD45 (clone 30F11; 1:30; Miltenyi Biotech), anti-CD31 (Clone 390; 1:30; Miltenyi Biotech), anti-CD11b (M1/70.15.11.5; 1:30; Miltenyi Biotech), and positive selection for APC-conjugated anti-Itgb1 (clone HMβ1-1; 1:15; Miltenyi Biotech) and PE-conjugated anti-Itga7 (clone 3C12, 1:100; Miltenyi Biotech), as previously reported (Dort et al., 2021). Myoblasts were seeded at 15% of confluency into 12-well plates, previously coated with collagen. Fresh media, consisting of Ham’s F10 media (GIBCO), 20% FBS (Wisent), 1% penicillin–streptomycin (GIBCO), and 2.5 ng/mL FGF2 (Wisent), was supplemented with RvD2 (200 nM), PSB-KD107 (10 μM), or vehicle (DMSO diluted in aqueous buffer), and added to cell culture. Cells were incubated in the IncuCyte (Essen Bioscience), which enables real-time counting of living myoblasts without influencing their function. Images were taken and processed automatically by the IncuCyte to determine myoblast proliferation through measurement of cell number and confluency over time. For the differentiation/fusion experiments, the cells at ∼80% confluence were incubated in low-serum medium containing a 1:1 ratio of HAM-F10 (Thermo Fisher scientific, #11550043) and DMEM (Wisent, #319-010-CL) with 5% horse serum. During the experimental protocol, the differentiation medium was supplemented with RvD2 (200 nM), PSB-KD107 (10 μM) or vehicle (DMSO diluted in aqueous buffer), from the first day of differentiation to the day 4 (medium renewed every 48 h). The fusion index was quantified as the proportion of nuclei located in multinucleated myotubes relative to the total nuclei count.

RvD2 was diluted in ethanol and stored at −80°C, while PSB-KD107 was diluted in DMSO and stored at −20°C. Both solutions were aliquoted, and the stock solution was thawed only once to avoid oxidation or degradation. To limit the injection of organic solvent, the RvD2 solution was evaporated, and the residue was resuspended in aqueous buffer as described by the manufacturer. For the PSB-KD107, the stock solution containing DMSO was diluted in aqueous buffer to obtain the working solution, according to the manufacturer instruction. Both solutions were made fresh every day and remaining solution was discarded after. Considering that the goal of this project was to study the efficacy of the synthetic Gpr18 analog, the vehicle was constituted of DMSO diluted in an aqueous buffer (like the PSB-KD107 solution). We could not add DMSO to the RvD2 solution as it could have isomerize and/or degrade this bioactive lipid. While this is a technical limitation of the study, our previous findings already shown that RvD2 was effective compared to its appropriate vehicle (Dort et al., 2021).

### RNA sequencing

Mdx myoblasts from 3 different mice treated for 16 h with PSB-KD107 or vehicle were harvested. The total mRNA was extracted using RNeasy Kit (Qiagen, ID:73,404) according to the manufacturer’s instructions, and was sent to Genome Quebec (Montreal, QC, Canada) for sequencing. Total RNA was quantified, and its integrity was assessed using 5K/RNA/Charge Variant Assay LabChip and RNA Assay Reagent Kit (Perkin Elmer). Libraries of RIN higher than 6.5 were generated from 250 ng of total RNA as following: mRNA enrichment was performed using the NEBNext Poly(A) Magnetic Isolation Module (New England BioLabs). cDNA synthesis was achieved with the NEBNext RNA First Strand Synthesis and NEBNext Ultra Directional RNA Second Strand Synthesis Modules (New England BioLabs). The remaining steps of library preparation were done using and the NEBNext Ultra II DNA Library Prep Kit for Illumina (New England BioLabs). Adapters and PCR primers were purchased from New England BioLabs. Libraries were quantified using the KAPA Library Quantification Kits - Complete kit (universal) (Kapa Biosystems). Average size fragment was determined using a LabChip GX II (PerkinElmer) instrument. The libraries were normalized and pooled and then denatured in 0.02N NaOH and neutralized using HT1 buffer. The pool was loaded at 200 p.m. on an Illumina NovaSeq S4 lane using Xp protocol as per the manufacturer’s recommendations. The run was performed for 2 × 100 cycles (paired-end mode). A phiX library was used as a control and mixed with libraries at 1% level. Base calling was performed with RTA v3. Program bcl2fastq2 v2.20 was then used to demultiplex samples and generate fastq reads. Sequencing data was processed on the Compute Canada server using an open-source Python-based Workflow Management System (GenPipes) (Bourgey et al., 2019). Quality control was performed using FastQC and MultiQC. Sequencing data were aligned to mouse reference genome m10 using STAR aligner version 2.3.0. Differentially expressed genes (DEGs) were identified using the Bioconductor package and DESeq2 package using Rstudio software. Based on read counts, significantly differentially expressed genes were identified based on a fold-change of two-fold or greater (up- or downregulated) and an adjusted *p*-value <0.05. An additional filter was put in place to remove genes in which the mean normalized Fragments Per Kilobase Mapped (FPKM) was <1 for both conditions to avoid changes in low abundance transcripts. To understand the biological impact of the gene expression changes and elucidate the functional annotation and pathway enrichment associated with the common DEGs, we performed Gene Ontology (GO) (Biological Process), GSEA (Gene Set Enrichment Analysis), and Kyoto Encyclopedia of Genes and Genomes (KEGG) pathway enrichment analysis using clusterProfiler package (Wu et al., 2021), and gProfiler server (Reimand et al., 2007). The enrichplot, DOSE (Yu et al., 2015) and Goplot packages (Walter et al., 2015) were used to supply enrichment result visualization and help interpretation.

### qPCR

Total RNA was extracted from the tibialis anterior muscle using TRIzol isolation technique. cDNA was synthesized using the All-In-One 5X RT MasterMix qPCR RT Kit (abm, #G592). Real time RT-qPCR was performed using a LightCycler^®^ 480 Instrument II with the BlasTaq 2X qPCR MasterMix (abm #G892). Primers used for the real time RT-qPCR are listed in Supplementary Table S1.

### Histology and immunofluorescence

Immunohistological staining was performed on cultured cells and skeletal muscle sections as previously described (Dumont and Rudnicki, 2017). For muscle sections, 10 μm thick sections were cut from the proximal and distal half of the TA muscle and mounted on Superfrost Plus slides (Thermo Fisher Scientific). The samples were fixed with 2% PFA (for immunofluorescence) or 4% PFA (for Sirius red) for 5 min and washed in distilled water. For Sirius red staining (Polysciences Inc.), the slides were incubated with Picrosirius Red solution for 90 min, rinsed with hydrochloride acid (0.1N) and distilled water for 2 min, dehydrated and mounted. For immunofluorescence, the fixed slides were incubated with permeabilization buffer (10 min in 0.2% Triton X-100, 0.1% glycine) at room temperature. Then, the slides were incubated with blocking buffer (90 min in 5% goat serum, and 2% BSA), followed by overnight incubation at 4°C with the following primary antibodies: mouse anti-Pax7 (clone PAX7, 1:20; Developmental Studies Hybridoma Bank (DSHB)), mouse anti-MyHCemb (clone F1.652; 0.3 μg/mL; DSHB), rabbit anti-Laminin (cat# ab11575; 1:1,000; Abcam), rat anti-F4/80 monoclonal antibody (clone A3-1; 1:1,000; Bio-Rad), rabbit CD206 monoclonal antibody (cat# ab64693; 1:2,000; Abcam), rat anti-Ly6G (clone 1A8, 1:150, Biolegend), mouse anti-myosin heavy chain (clone MF20; 1:20; DSHB), rabbit anti-myogenin (clone EPR4789; 1:500; Abcam), mouse anti-Pax3 (2 μg/mL; DSHB), goat anti-T (human Brachyury, #AF 2085, R&D system), mouse anti-OCT4 (clone 3A2A20, Biolegend), rabbit anti-SSEA4 (#MBS460512, MyBiosource). Thereafter, samples were washed, and incubated for 1 h with secondary antibodies (Thermo Fisher): goat anti-rabbit IgG H + L (Alexa Fluor 488, 1:1,000), goat anti-mouse IgG1 (Alexa Fluor 546, 1:1,000), goat anti-rat IgG H + L (Alexa Fluor 594, 1:1,000), goat anti-mouse IgG H + L (Alexa Fluor 488, 1:1,000), or donkey anti-goat IgG H + L (Alexa Fluor 488, 1:1,000). Samples were then incubated with DAPI for 5 min, washed, mounted with PermaFluor (Thermo Fisher Scientific), and imaged with an epifluorescence Leica DM5000 B (Leica Microsystems, Canada) or EVOS M5000 (Thermo Fisher Scientific). A minimum of 5 images were randomly acquired from proximal and distal portion of the muscle for analysis. For Sirius Red staining, the slides were visualized under polarized light, which provides a more precise and clear identification of the collagen network (Rittié, 2017). The proportion of Sirius red staining relative to the entire muscle area was quantified by ImageJ software (National Institutes of Health, USA). For the assessment of muscle fiber cross-sectional area, the sections were analyzed using the MuscleAnalyzer pipeline on the CellProfiler software (Lau et al., 2018), and a minimum of 500 myofibers were analyzed.

## Western blot

Total protein was extracted by lysing cells with RIPA buffer containing 1% of protease inhibitors, and then quantified using the BCA assay kit (Thermo Scientific) according to the manufacturer’s protocol. Western blot was performed on 50 μg of protein suspension that was diluted in sample buffer (125 mM Tris buffer (pH6.8), 4% SDS, 20% glycerol, 0.05% bromophenol blue, and 200 mM dithiothreitol), denatured at 100 °C for 5 min, loaded and separated on a 9% sodium dodecyl sulfate-polyacrylamide gel. A 5 μl of Precision Plus Protein Dual Color Standards (Bio-Rad Laboratories) was added in a separate well as a molecular weight marker. Samples were transferred to polyvinylidene difluoride membranes, which were blocked with 5% BSA for 1 h at room temperature. Membranes were then incubated overnight at 4°C with the primary antibodies: rabbit anti-phospho-Akt (Ser473) (cat# 9271; 1:1,000 in 5% BSA; Cell Signaling Technology), rabbit anti-(pan)-Akt (clone C67E7; 1:1,000 in 5% BSA; Cell Signaling Technology), rabbit anti-Gpr18 (cat# ab76258; 1:1,000 in 5% non-fat milk; Abcam), rabbit anti-beta-actin (cat# 4967; 1:1,000 in 5% non-fat milk; Cell Signaling Technology), or rabbit anti-GAPDH (cat# 2118; 1:1,000 in 5% BSA; Cell Signaling Technology). Thereafter, membranes were serially washed, and incubated with the goat anti-rabbit (H + L) HRP-conjugated secondary antibody (1:3,000; Abcam) for 1 h at room temperature, allowing the detection of specific bands revealed with ECL-plus Western blotting reagent (PerkinElmer Life and Analytical Sciences, USA). Signals were captured with G:BOX using the GeneSys image software (Syngene).

### Force measurement

Muscle function was assessed *ex vivo* as previously described (Dort et al., 2021). Briefly, the extensor digitorum longus muscle was carefully harvested and incubated in Krebs-Ringer solution supplemented with glucose and carbogen, and maintained at 25 °C. The proximal and distal tendons were respectively attached to the electrode and the lever arm (300C-LR dual-mode lever; Aurora Scientific, Canada). Thereafter, the muscle tension was adjusted to find the optimal length (i.e., length at which the muscle generates a maximal twitch force). After 10 min equilibration, muscles were stimulated at different frequencies (10-150 Hz) with a 2-min rest between each stimulation to obtain a force-frequency curve. Muscle length and weight were recorded to assess specific muscle force (N/cm^2^), as previously reported (Dort et al., 2021).

### Statistical analysis

Data was analyzed using the GraphPad Prism 9 Software, and the following tests were used as appropriate: Student’s t-test, One-way or Two-way analysis of variance (ANOVA) followed by Tukey or uncorrected Fisher’s Least Significant Difference test (the test used are specified in figure legends). Statistical significance was determined with 95% confidence intervals, and a *p*-value <0.05. Results are reported as mean ± standard error of the mean (SEM). *p < 0.05, **p < 0.01, ***p < 0.001, ****p < 0.0001.

## Data Availability

The datasets presented in this study can be found in online repositories (Gene Expression Omnibus database, accession number GSE222773). The names of the repository/repositories and accession number(s) can be found in the article/Supplementary Material.
