# Supplementary material for: Gpr18 agonist dampens inflammation, enhances myogenesis, and restores muscle function in models of Duchenne muscular dystrophy
Source: Front Cell Dev Biol. 2023 Aug 14;11:1187253. doi: 10.3389/fcell.2023.1187253 (PMC10461444; doi:10.3389/fcell.2023.1187253)
Supplement: Supplementary file 1 [file DataSheet1.PDF]

Supplementary information

for

Gpr18 agonist dampens inflammation, enhances myogenesis, and restores muscle function in models of Duchenne Muscular Dystrophy.

Junio Dort, Zakaria Orfi, Melissa Fiscaletti, Philippe M. Campeau, and Nicolas A. Dumont

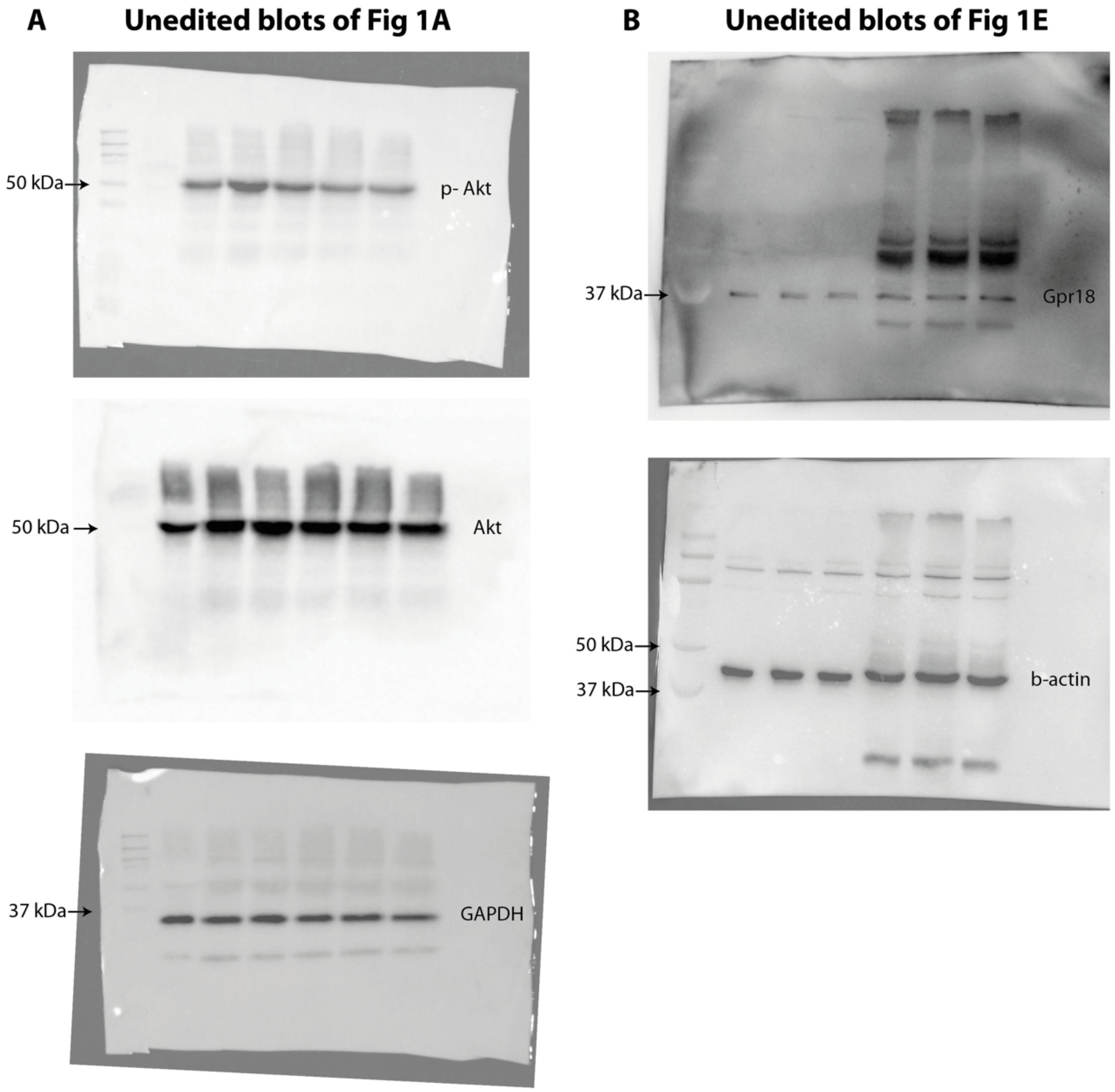

Supplementary figure 1: Unedited gels. Full unedited gels of the Western Blot experiments for **A**) figure 1A and **B**) figure 1E.

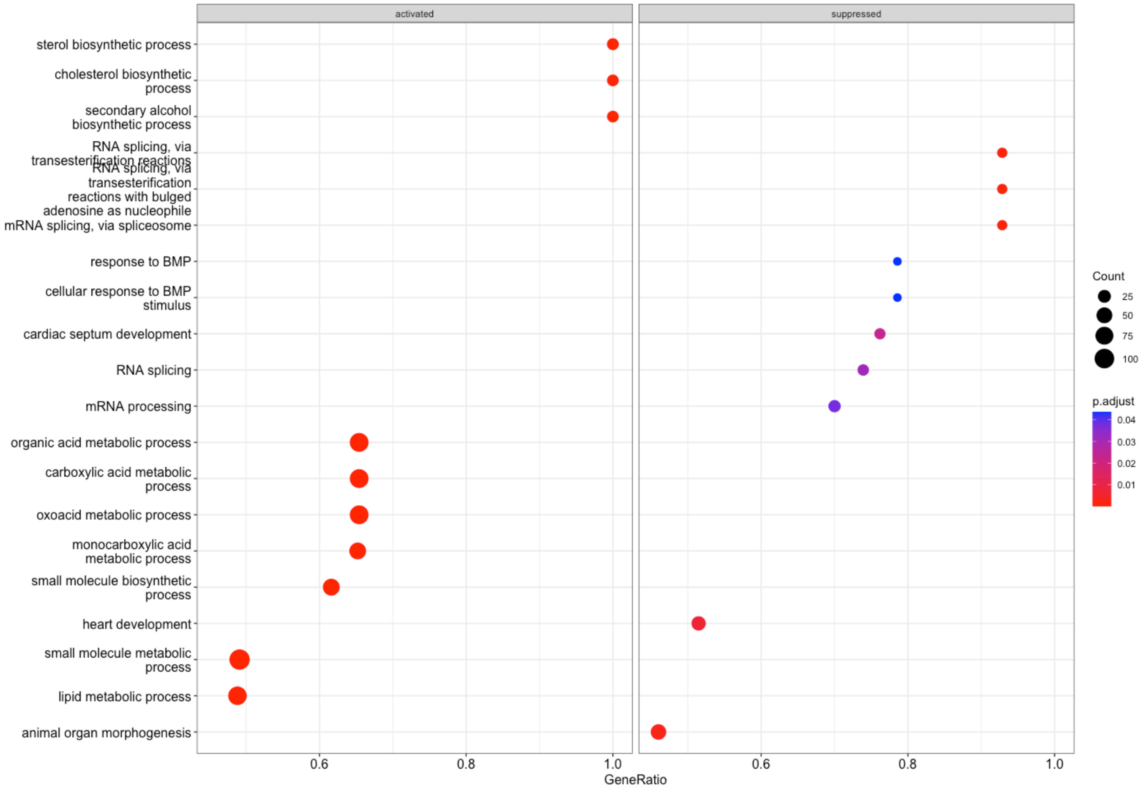

Supplementary figure 2: RNAseq analysis. GO term analysis showing the enriched biological processes for the top upregulated genes (left column) and downregulated genes (right column) in PSB-KD107-treated mdx myoblasts vs vehicle-treated cells.

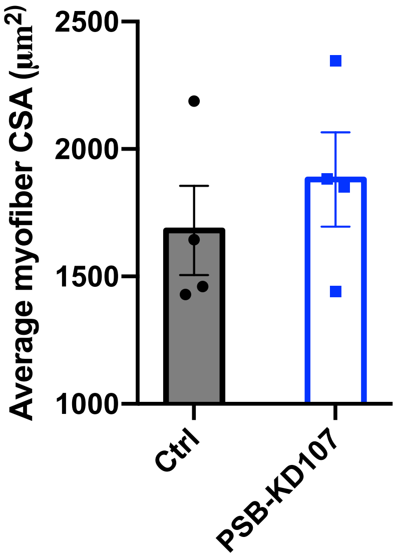

Supplementary figure 3. Mean myofiber diameter. Mdx mice were treated with weekly injections of PSB-KD107 (1 mg/kg) or vehicle for 3 weeks (Ctrl). Quantification of mean myofiber cross-sectional area (CSA) in the TA muscle of PSB-KD107 or Ctrl mice. N=4. Results shown as mean +/- SEM.

Gene	Forward sequence	Reverse sequence
Cd80	TGCTCTCAGAACCAAGCCAC	ATGCTGCAGCTTACTTCCCC
Gpr18	CTGAAGCCCAAGGTCAAGGA	TTGTAGCATCAGGACGGCAA
Ptgs2	CATCCCCTTCCTGCGAAGTT	CATGGGAGTTGGGCAGTCAT
Anxa1	GGTGACCGTTGTCAGGACTT	CTGGTGGCACACTTCACGAT
Gapdh	CCCAGAAGACTGTGGATGG	ACACATTGGGGGTAGGAACA

Supplemental table 1. List of primers used for qPCR experiments
